# Correction to “Sustainable In-Water Synthesis of Aliphatic Porous Polyazines: A Versatile Platform for Conjugated Aerogels, PolyHIPEs, or Carbon Foams”

**DOI:** 10.1021/acs.macromol.4c01406

**Published:** 2024-07-11

**Authors:** Tomaž Kotnik, Gregor Žerjav, Zoran Novak, Albin Pintar, Sebastijan Kovačič

**Affiliations:** † National Institute of Chemistry, Hajdrihova 19, SI-1001 Ljubljana, Slovenia; ‡ Faculty of Chemistry and Chemical Technology, University of Ljubljana, 1000 Ljubljana, Slovenia; § Faculty of Chemistry and Chemical Engineering, University of Maribor, Smetanova 17, SI-2000 Maribor, Slovenia

The second affiliation of Tomaž Kotnik was not included and needs to be added. The correct affiliation is as follows:

Tomaž Kotnik - National Institute of Chemistry, Hajdrihova 19, SI-1001 Ljubljana, Slovenia; Faculty of Chemistry and Chemical Technology, University of Ljubljana, 1000 Ljubljana, Slovenia

The change is reflected in the Author Information section of this Correction, while the content and conclusions of this publication remain unaffected.

